# Morphine and Clonidine Combination Therapy Improves Therapeutic Window in Mice: Synergy in Antinociceptive but Not in Sedative or Cardiovascular Effects

**DOI:** 10.1371/journal.pone.0109903

**Published:** 2014-10-09

**Authors:** Laura S. Stone, Jonathan P. German, Kelly F. Kitto, Carolyn A. Fairbanks, George L. Wilcox

**Affiliations:** 1 Alan Edwards Centre for Research on Pain, McGill University, Montreal, Quebec, Canada; 2 Faculty of Dentistry, McGill University, Montreal, Quebec, Canada; 3 Department of Anesthesiology, Faculty of Medicine, McGill University, Montreal, Quebec, Canada; 4 Department of Pharmacology and Therapeutics, Faculty of Medicine, McGill University, Montreal, Quebec, Canada; 5 Department of Neurology and Neurosurgery, Faculty of Medicine, McGill University, Montreal, Quebec, Canada; 6 Department of Pharmaceutics, College of Pharmacy, University of Minnesota, Minneapolis, Minnesota, United States of America; 7 Department of Neuroscience, Medical School, University of Minnesota, Minneapolis, Minnesota, United States of America; 8 Department of Pharmacology, Medical School, University of Minnesota, Minneapolis, Minnesota, United States of America; 9 Department of Dermatology, Medical School, University of Minnesota, Minneapolis, Minnesota, United States of America; Temple University, United States of America

## Abstract

Opioids are used to manage all types of pain including acute, cancer, chronic neuropathic and inflammatory pain. Unfortunately, opioid-related adverse effects such as respiratory depression, tolerance, physical dependence and addiction have led to an underutilization of these compounds for adequate pain relief. One strategy to improve the therapeutic utility of opioids is to co-administer them with other analgesic agents such as agonists acting at α_2_-adrenergic receptors (α_2_ARs). Analgesics acting at α_2_ARs and opioid receptors (ORs) frequently synergize when co-administered *in vivo*. Multimodal analgesic techniques offer advantages over single drug treatments as synergistic combination therapies produce analgesia at lower doses, thus reducing undesired side effects. This inference presumes, however, that the synergistic interaction is limited to the analgesic effects. In order to test this hypothesis, we examined the effects of α_2_AR/OR combination therapy in acute antinociception and in the often-undesired side effects of sedation and cardiovascular depression in awake unrestrained mice. Morphine, clonidine or their combination was administered by spinal or systemic injection in awake mice. Antinociception was determined using the warm water tail flick assay (52.5°C). Sedation/motor impairment was evaluated using the accelerating rotarod assay and cardiovascular function was monitored by pulse oximetry. Data were converted to percent maximum possible effect and isobolographic analysis was performed to determine if an interaction was subadditive, additive or synergistic. Synergistic interactions between morphine and clonidine were observed in the antinociceptive but not in the sedative/motor or cardiovascular effects. As a result, the therapeutic window was improved ∼200-fold and antinociception was achieved at non-sedating doses with little to no cardiovascular depression. In addition, combination therapy resulted in greater maximum analgesic efficacy over either drug alone. These data support the utility of combination adrenergic/opioid therapy in pain management for antinociceptive efficacy with reduced side-effect liability.

## Introduction

Opioid receptor agonists have analgesic properties following both spinal and systemic administration [1], [2]. Opioid analgesics remain the mainstay for the treatment of moderate to severe pain [3]. However, the utility of opioid analgesics is limited by the incidence and prevalence of well-known problematic effects, including respiratory and cardiovascular depression [4], sedation, constipation, nausea, cognitive impairment, itch, and the development of analgesic tolerance [1].

Agonists acting at α_2_ARs have analgesic properties in multiple species including humans [5]–[15]. Therapeutic development of α_2_AR agonists for the treatment of pain is particularly important for the management of patients who are under-responsive to conventional opioid therapy [10], [12], [16]–[23]. The prototypic α_2_AR agonist, clonidine, is currently approved for spinal delivery in intractable cancer pain. However, the therapeutic utility of α_2_AR agonists has been hampered by their side-effect profile, with sedation and hypotension being of particular concern [24]–[26].

Co-administration of α_2_AR agonists with opioids often results in a greater-than-additive (i.e. synergistic) interaction following either spinal or systemic delivery [27]–[36], although the interaction is of greater magnitude in the spinal cord [28], [29]. Synergistic drug interactions result in enhanced potency and/or efficacy when one agent is given together with another. Therapeutic application of synergistic adrenergic-opioid combinations is important in pain management because of the expectation of improved efficacy and reduced doses, and theoretically, reduced side effects [13], [25], [37]. This inference presumes, however, that the synergistic interaction is limited to the desired analgesic effect and not the undesired side effect(s), which may not always be the case [38]. The objective of the current study is to address this presumption.

The potential for adrenergic-opioid combination therapy to improve clinical utility depends on the potentiation of analgesia without similar potentiation of the side effects. The effects of co-administered morphine and clonidine on antinociception, sedation/motor impairment, heart rate and a surrogate of blood pressure were examined to determine if combination therapy could be used to increase the therapeutic window. The present study therefore assessed effects on these variables in unrestrained, awake, behaving mice to test for both sedative/motor and cardiovascular side effects of adrenergic-opioid combination therapy.

## Methods

### Animals

Male CD-1 ICR mice (20±5 g; Harlan, Madison, WI) were maintained on a 12-hour light/dark cycle with unlimited access to food and water. All experiments were approved by the Institutional Animal Care and Use Committee of the University of Minnesota (Permit #0407A62285) and conformed to the ethical guidelines of the Guide for the Care and Use of Laboratory Animals of the National Institutes of Health and the guidelines of the Committee for Research and Ethical Issues of the International Association for the Study of Pain [39].

### Drug Preparation and Administration

Morphine sulfate (NIDA) and clonidine (Sigma; St. Louis, MO) were dissolved in 0.9% saline. Intrathecal (i.t.) drug administration was done by direct lumbar puncture in a volume of 5 µL according to the method of Hylden and Wilcox in conscious mice [2]. Following i.t. administration, tail flick latencies, heart rate and carotid distension were obtained at 10 and 30 minutes and rotarod retention times were obtained 15 and 35 minutes post-injection. Intraperitoneal injections (i.p.) were administered in a total volume of 100 µL per 25 g. Following i.p. administration, tail flick latencies, heart rate and carotid blood flow were obtained 15 and 60 minutes and rotarod retention times were obtained 20 and 65 minutes post-injection. The tail flick and rotarod assays were performed in the same animals sequentially. The cardiovascular measures were obtained in a separate set of mice.

### Antinociception

Antinociception was assessed using the warm water (52.5°C) tail immersion assay [40]. Mice were gently wrapped in a soft cloth such that their tails were exposed and three quarters of the length of each tail was dipped into the hot water. Tail flick latencies were obtained before and after drug administration. A maximum cut-off of 12 seconds was set in order to avoid tissue damage. A minimum of three mice were used for each dose. The results are expressed as a percent of the maximum possible effect (%MPE) according to the equation: 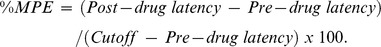



### Sedation/Motor Impairment

Retention time on an accelerating rotorod was used as a model of motor coordination and/or sedation, since sedation will also cause the animals to fall. Animals were trained until retention times exceeded 240 seconds. A maximum cutoff of 5 min was used. The results are expressed as a percent of the maximum possible effect (%MPE) according to the equation: 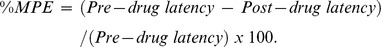



### Cardiovascular Measurements

Cardiovascular function was monitored in awake, freely moving mice trained one day prior to tolerate the presence of a pulse oximetry clip on the dorsal half of the neck (MouseOx, Starr Life Sciences Corp). Because albino ICR mice were used in this study, pulse oximetry of the carotid arteries was possible without shaving. Carotid distension and heart rate were recorded before and after drug or combination administration. Carotid distension (µm) is an indicator of carotid blood flow and was used as a surrogate for blood pressure. Maximum efficacy was set at 300 beats/minute for heart rate and 300 µm for carotid distension. Oxygen saturation remained between 97-100% regardless of treatment. The results are expressed as a percent of the maximum possible effect (%MPE) according to the equation: 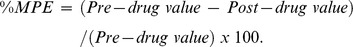



### Dose-Response Analysis

Individual dose points are expressed as means with standard error of the mean (SEM). ED_50_ values and confidence limits were calculated according to the graded dose-response method of Tallarida and Murray [41] on the linear portion of each dose-response curve. A minimum of three doses were used for each drug or combination of drugs.

### Therapeutic Window

The therapeutic window (TW) was estimated using the ratio: TW  =  (ED_50_ value for sedation, heart rate or carotid distension)/(ED_50_ value for antinociception).

### Isobolographic Analysis

Isobolographic analysis is the ‘gold standard’ for evaluating drug interactions [41], [42]. Dose-response curves were constructed for each agonist administered alone, the ED_50_ values were calculated and used to determine an equieffective dose ratio between the agonists. This ratio was then maintained when both agonists were administered in combination, a third dose-response curve was constructed and an experimentally derived combination ED_50_ was calculated. To test for interactions between agonists, the ED_50_ values and standard error of all dose-response curves were arithmetically arranged around the ED_50_ value using the following equation: 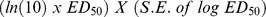

[42]. Isobolographic analysis necessitates this manipulation. When testing an interaction between two drugs, a theoretical additive ED_50_ value is calculated for the combination based on the dose-response curves of each drug administered separately. This theoretical value is then compared by a *t*-test with the observed experimental ED_50_ value of the combination. These values are based on the total dose of both drugs. An interaction is considered synergistic if the experimental ED_50_ is significantly less (p<0.05) than the calculated theoretical additive ED_50_.

**Figure 1:**
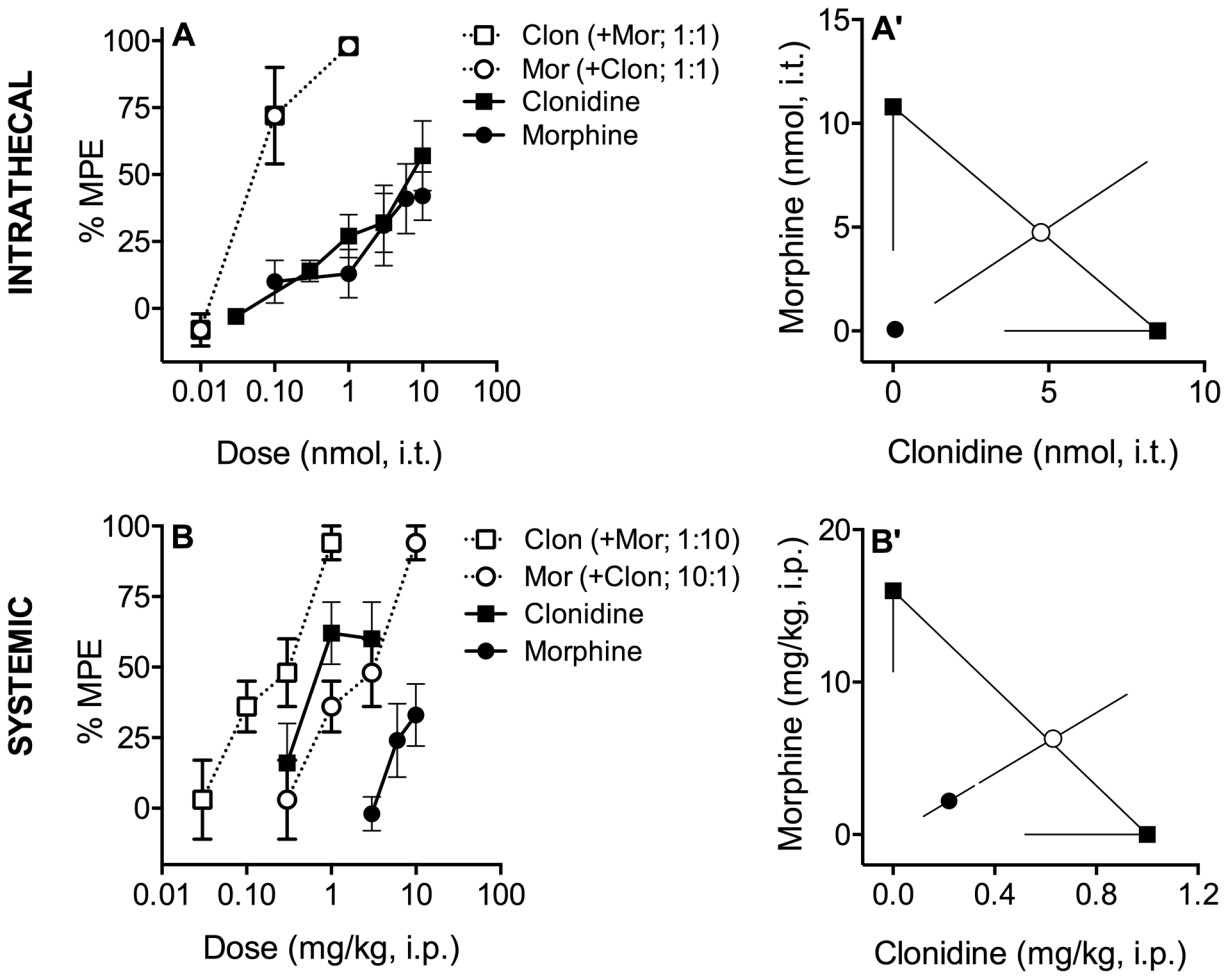
Effects of Morphine and Clonidine in the Tail Flick Antinociception Assay. **A**: Intrathecally delivered morphine (•) and clonidine (▪) dose-dependently inhibited thermal nociception with similar potency and efficacy. When co-administered at a constant dose ratio of 1∶1 (○ morphine; □ clonidine), both potency and efficacy were increased. **A**′: Isobolographic analysis applied to the data from Figure 1A. The y-intercept represents the ED_50_ for morphine and the x-intercept represents the ED_50_ for clonidine. The lines directed from each ED_50_ value toward zero represent the lower 95% confidence limits of each ED_50_. The line connecting these two points is the theoretical additive line. The unfilled circle on the theoretical additive line represents the calculated theoretical ED_50_ value of the combination if the interaction is additive. The observed combination ED_50_ (•) was significantly (p<0.05; t-test) lower than the theoretical additive ED_50_ (○), indicating that the interaction is synergistic. **B**. Systemically administered morphine (•) and clonidine (▪) dose-dependently inhibited thermal nociception when administered either alone or in combination at a constant morphine∶clonidine dose ratio of 10∶1 (○ morphine; □ clonidine). **B**′: Isobolographic analysis applied to the data from Figure 1B. The y-intercept represents the ED_50_ for morphine and the x-intercept represents the ED_50_ for clonidine. The observed combination ED_50_ (•) was significantly (p<0.05; t-test) lower than the theoretical additive ED_50_ (○), indicating that the interaction is synergistic. Data pictured were obtained 10 minutes following intrathecal (Figures 1A, A′) and 15 minutes following systemic administration (Figures 1B, B′). Error bars represent ±SEM for each dose point (*n* = 6–10 animals/dose). See Table 1 for ED_50_ values.

**Figure 2:**
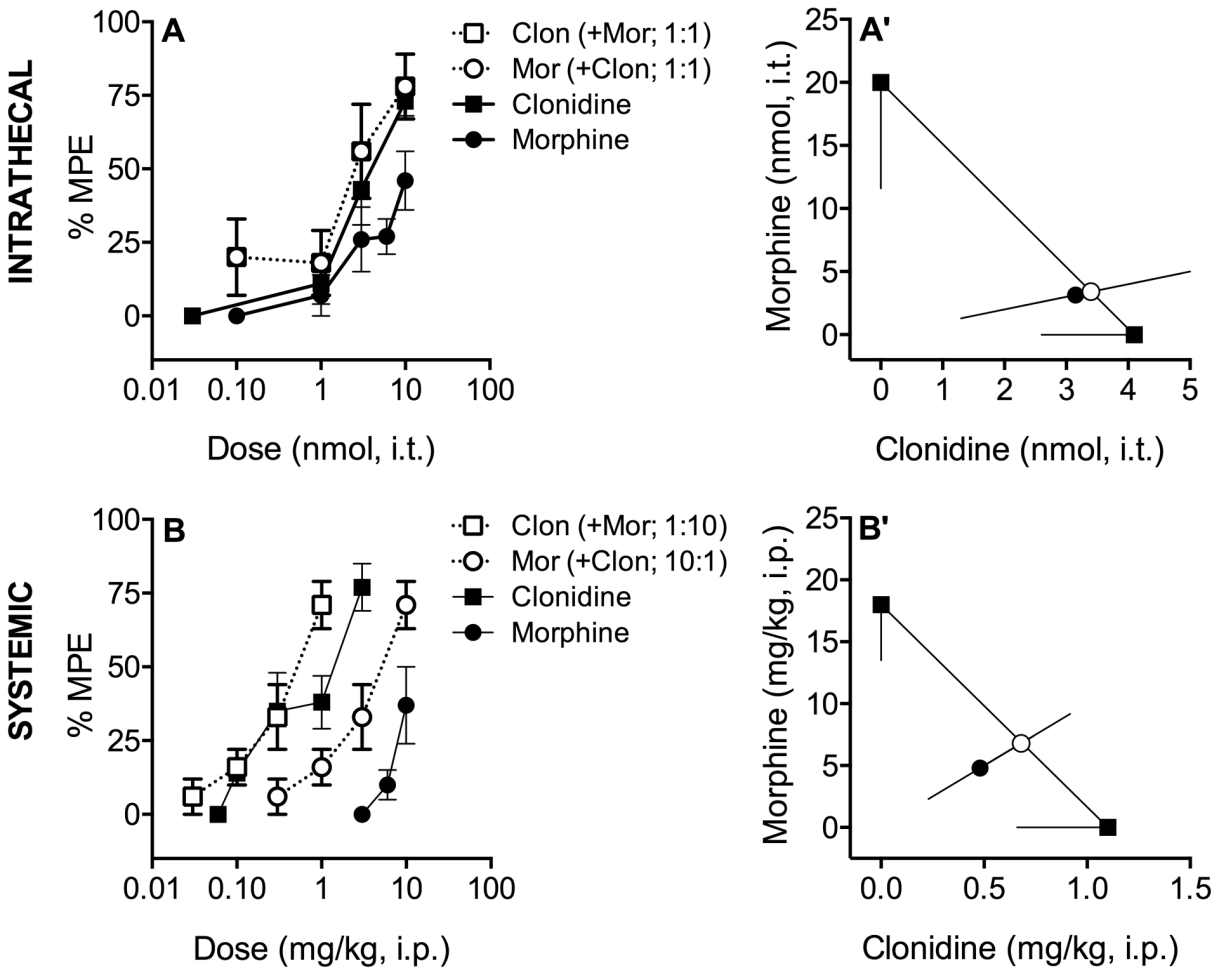
Effects of Morphine and Clonidine in the Rotarod Sedation Assay. **A**. Rotarod retention was challenged by intrathecal morphine, clonidine or both. Morphine (•) and clonidine (▪) inhibited rotarod performance in a dose-dependent manner with similar potency. When the agonists were co-administered at a constant ratio of 1∶1 (○ morphine; □ clonidine), no increases in potency or efficacy were observed. **A**′. Isobolographic analysis applied to the data from Figure 2A. The y-intercept represents the ED_50_ for morphine and the x-intercept represents the ED_50_ for clonidine. The lines directed from each ED_50_ value toward zero represent the lower 95% confidence limits of each ED_50_. The line connecting these two points is the theoretical additive line. The unfilled circle on the theoretical additive line represents the calculated theoretical ED_50_ value of the combination if the interaction is additive. The observed combination ED_50_ (•) is not significantly (p<0.05; t-test) different from the theoretical additive ED_50_ (○), indicating that the interaction is additive. **B**. Systemically administered morphine (•) and clonidine (▪) dose-dependently inhibited rotarod performance when administered alone or in combination at a constant morphine∶clonidine dose ratio of 10∶1 (○ morphine; □ clonidine). **B**′. Isobolographic analysis applied to the data from Figure 2B. The observed combination ED_50_ (•) is not significantly (p<0.05; t-test) different than the theoretical additive ED_50_ (○), indicating that the interaction is additive. Data pictured were obtained 15 minutes following intrathecal (Figures 2A, A′) and 20 minutes following systemic administration (Figures 2B, B′). Error bars represent ±SEM for each dose point (*n* = 6–10 animals/dose). See Table 1 for ED_50_ values.

**Figure 3:**
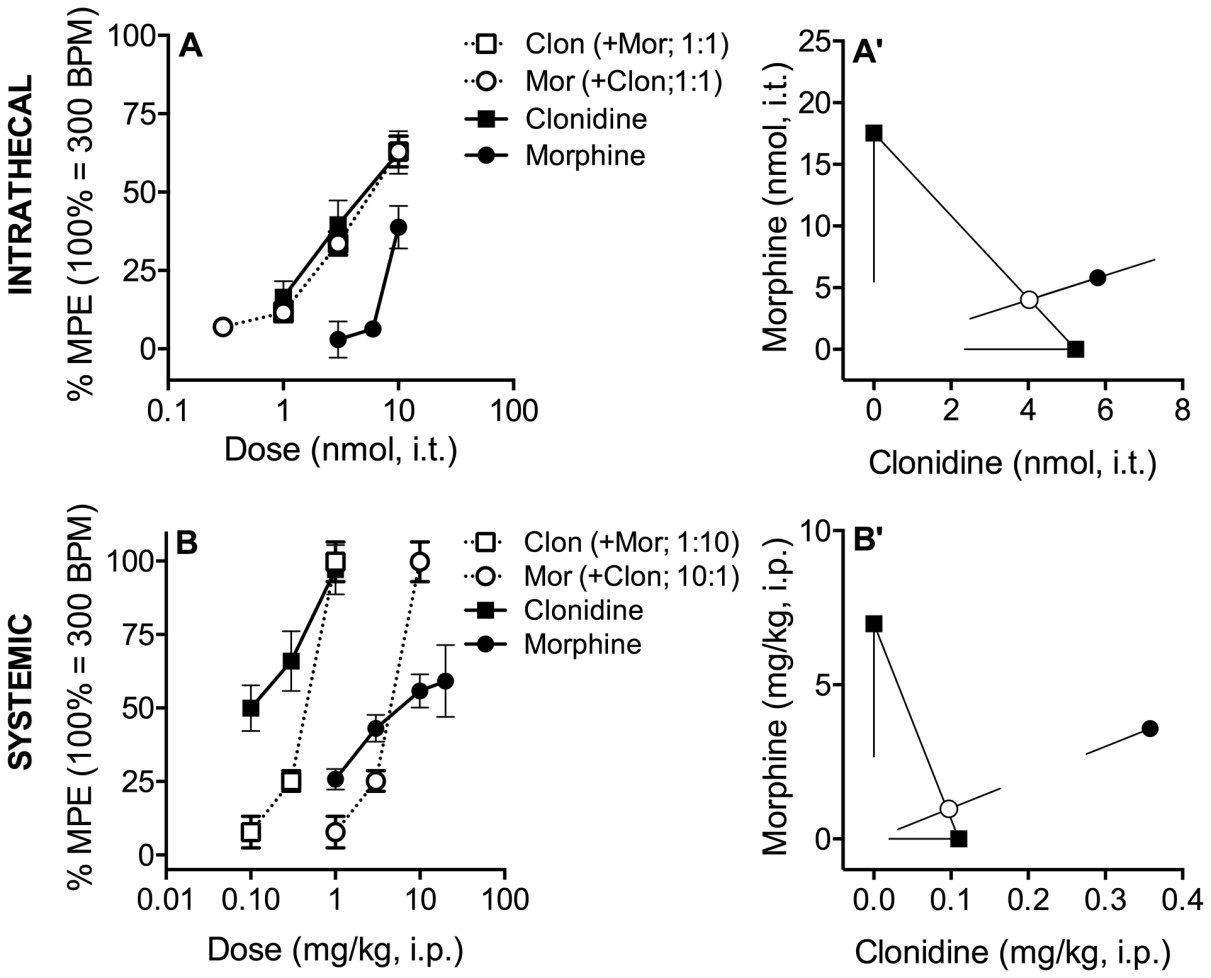
Effects of Morphine and Clonidine on Heart Rate. **A**. Heart rate was challenged by intrathecal morphine, clonidine or both. Morphine (•) and clonidine (▪) inhibited heart rate in a dose-dependent manner. When the agonists were co-administered at a constant ratio of 1∶1 (○ morphine; □ clonidine), no increases in potency or efficacy were observed. **A**′. Isobolographic analysis applied to the data from Figure 3A. The y-intercept represents the ED_50_ for morphine and the x-intercept represents the ED_50_ for clonidine. The lines directed from each ED_50_ value toward zero represent the respective lower 95% confidence limits of each ED_50_. The line connecting these two points is the theoretical additive line. The unfilled circle on the theoretical additive line represents the calculated theoretical ED_50_ value of the combination if the interaction is additive. The observed combination ED_50_ (•) is not significantly (p<0.05; t-test) different from the theoretical additive ED_50_ (○), indicating that the interaction is additive. **B**. Systemically administered morphine (•) and clonidine (▪) dose-dependently inhibited heart rate when administered alone or in combination at a constant morphine∶clonidine dose ratio of 10∶1 (○ morphine; □ clonidine). **B**′. Isobolographic analysis applied to the data from Figure 3B. The observed combination ED_50_ (•) is significantly (p<0.05; t-test) higher than the theoretical additive ED_50_ (○), indicating that the interaction is sub-additive. Data pictured were obtained 10 minutes following intrathecal (Figures 3A, A′) and 15 minutes following systemic administration (Figures 3B, B′). Error bars represent ±SEM for each dose point (*n* = 6–10 animals/dose). See Table 1 for ED_50_ values. *Note: 100% MPE was artificially set at 300 beats per minute (baseline ∼800) to facilitate dose-response and isobolographic analysis*.

Visualization of drug interactions can be facilitated and enhanced by graphical representation of isobolographic analysis (Figures 1–3, A′ and B′). This representation depicts the ED_50_ of each agent as the x- or y-intercept. For example, Figure 1A′ presents the ED_50_ of morphine as the y-intercept and the ED_50_ of clonidine as the x-intercept. The line connecting these two points depicts the dose combinations expected to yield 50% efficacy if the interaction is purely additive and is called the theoretical additive line. The theoretical additive ED_50_ and its confidence interval are determined mathematically and plotted spanning this line. The observed ED_50_ for the combination is plotted at the corresponding x,y co-ordinates along with its 95% confidence interval for comparison to the theoretical additive ED_50_.

### Interaction Index

The magnitude of drug interactions can be expressed in terms of an Interaction Index (γ) [43]. The index is defined by the equation: a/A + b/B  =  γ, where A and B are the doses of drugs A and B alone that give a specified level of effect and (a,b) is the combination dose that produces this same level of effect. In the absence of a drug interaction, γ = 1. If the interaction is synergistic, γ<1. The interaction index is used here as a quantitative measure to characterize the magnitude of the interactions afforded by a combination; smaller γ indicates a more profound synergistic interaction.

All dose-response and isobolographic analyses were performed with the FlashCalc pharmacological statistics software package generously supplied by Dr. Michael Ossipov. Figures were prepared using Graphpad Prism 6.0.

## Results

### Intrathecal co-administration of morphine and clonidine has a synergistic effect on antinociception

Intrathecal administration of either morphine or clonidine produced dose-dependent antinociception at 10 (Figure 1A, Table 1) and 30 minutes post-injection (Table 1) at a potency ratio of approximately 1∶1 morphine∶clonidine.

**Table 1:** Effect of Combination Therapy on Drug Potency.

Assay	Route	Time (min)	Morphine ED_50_ (±SEM)	Clonidine ED_50_ (±SEM)	Observed combination ED_50_ (±SEM)	Theoretical combination ED_50_ (±SEM)	γ	Interaction
**Tail Flick**	**Spinal (nmol)**	**10**	*∼11 (±11)*	8.5 (±7.2)	0.14 (±0.12)	9.5 (±6.8)	0.02	**Synergistic**
		**30**	5.3 (±3.3)	2.5 (±1.4)	0.25 (±0.16)	3.4 (±1.5)	0.07	**Synergistic**
	**Systemic (mg/kg)**	**15**	*∼16 (±7)*	1.0 (±0.7)	2.4 (±1.1)	6.9 (±3.2)	0.4	**Synergistic**
		**60**	*∼7.8 (±4.6)*	1.0 (±0.7)	1.6 (± 0.8)	4.9 (±2.2)	0.3	**Synergistic**
**Rotarod**	**Spinal (nmol)**	**15**	*∼20 (±11)*	4.1 (±1.9)	6.3 (±3.7)	6.8 (±2.9)	0.9	**Additive**
		**35**	*∼30 (±10)*	*∼9.4 (±6.3)*	*∼50 (±60)*	14 (±7.4)	3.5	**Additive**
	**Systemic (mg/kg)**	**20**	*∼18 (±6)*	1.1 (±0.6)	*5.3 (±2.8)*	7.5 (±2.6)	0.7	**Additive**
		**65**	*No efficacy*	2.4 (±1.2)	11 (±9)	*NA*	*NA*	*No interaction
**Heart Rate**	**Spinal (nmol)**	**10**	*∼18 (±12)*	5.2 (±2.4)	12 (±3)	8.1 (±3.1)	1.4	**Additive**
		**30**	*∼14 (±8)*	5.3 (±2.1)	13 (±5)	7.6 (±2.5)	1.7	**Sub-additive**
	**Systemic (mg/kg)**	**15**	7.0 (±4.3)	0.11 (±0.09)	3.9 (±0.9)	1.1 (±0.7)	3.8	**Sub-additive**
		**60**	4.6 (±1.7)	0.27 (±0.09)	2.9 (±0.6)	1.9 (±0.5)	1.6	**Sub-additive**
**Carotid Distension**	**Spinal (nmol)**	**10**	*No efficacy*	5.4 (±3.1)	5.0 (±2.4)	*NA*	*NA*	*No interaction
		**30**	*No efficacy*	*∼10 (±8)*	*∼11 (±7)*	*NA*	*NA*	*No interaction
	**Systemic (mg/kg)**	**15**	*No efficacy*	0.21 (±0.09)	0.45 (±0.20)	*NA*	*NA*	*No interaction
		**60**	*No efficacy*	0.68 (±0.59)	*No efficacy*	*NA*	*NA*	*NA*

Morphine and clonidine were administered in a dose-ratio of 1∶1 for spinal and 10∶1 for systemic administration; ∼ indicates that the ED_50_ value was determined by extrapolation (in cases where maximum efficacy was between 30 and 50%); γ  =  Interaction index (an index of 1 =  no interaction, index >1 =  potentiation, index <1  =  antagonism); * indicates that the ED_50_ value of clonidine alone was not significantly different from that of clonidine in the presence of morphine; NA  =  not available (it is not possible to calculate these values).

Co-administration of morphine and clonidine at a dose ratio equal to the potency ratio (1∶1) resulted in an ∼100-fold increase in potency, suggesting that the interaction is synergistic. If the interaction were additive, the potency of the combination would have increased by ∼2-fold. The dose-response data from Figure 1A are presented in an isobologram in Figure 1A′. As shown in Figure 1A′, the ED_50_ of the combination (closed circle) is lower than the theoretical additive ED_50_ (open circle), indicating that this interaction is synergistic. Similar results were obtained at 10- and 30-minutes post-treatment (Table 1). The interaction index, γ, was 0.02 and 0.07 at these two time points, respectively. Since smaller γ values indicate increasing levels of synergism, these values indicate that the synergistic interaction between morphine and clonidine is profound.

When administered alone, neither morphine nor clonidine reached full efficacy (defined as ≥75%) 10 minutes following injection (Figure 1A, Table 2). In contrast, 100% efficacy was achieved by the combination (Table 2).

**Table 2:** Effect of Combination Therapy on Drug Efficacy.

Assay	Route	Time (min)	Morphine Max (±SEM)	Clonidine Max (±SEM)	Mor+ Clon Max (±SEM)
**Tail Flick**	**Spinal**	**10**	42 (±9)	57 (±13)	**99 (±0.9)**
		**30**	69 (±15)	84 (±9)	**100 (±0)**
	**Systemic**	**15**	33 (±11)	62 (±11)	**94 (±6.0)**
		**60**	48 (±13)	76 (±15)	**100 (±0)**
**Rotarod**	**Spinal**	**15**	46 (±10)	**78 (±5)**	73 (±5)
		**35**	34 (±11)	**48 (±12)**	43 (±15)
	**Systemic**	**20**	37 (±13)	**77 (±8)**	71 (±8)
		**65**	21 (±11)	**66 (±13)**	48 (±12)
**Heart Rate**	**Spinal**	**10**	39 (±7)	**63 (±7)**	**63 (±5)**
		**30**	47 (±4)	**73 (±9)**	63 (±6)
	**Systemic**	**15**	59 (±12)	97 (±8)	**100 (±7)**
		**60**	69 (±2)	92 (±14)	**113 (±7)**
**Carotid Distension**	**Spinal**	**10**	12 (±8)	58 (±8)	**62 (±6)**
		**30**	9.4 (±2.2)	**48 (±9)**	43 (±5)
	**Systemic**	**15**	7.3 (±3.1)	**88 (±8)**	72 (±18)
		**60**	9.5 (±4.5)	**63 (±19)**	27 (±9)

Max  =  maximum efficacy, the drug or drug combination that achieved the maximum efficacy is indicated in bold, BPM  =  beats per minute.

### Systemic co-administration of morphine and clonidine has a synergistic effect on antinociception

Systemic (i.p.) administration of either morphine or clonidine produced dose-dependent antinociception 15 (Figure 1B, Table 1) and 60 minutes (Table 1) at a potency ratio of approximately 10∶1 morphine∶clonidine.

Co-administration of morphine and clonidine at a dose ratio equal to the potency ratio (10∶1) resulted in an ∼10-fold increase in potency, suggesting that the interaction is synergistic (Figure 1B, Table 1). As shown in Figure 1B′, the ED_50_ of the combination (closed circle) is lower than the theoretical additive ED_50_ (open circle), indicating that the interaction is synergistic. The interaction index, γ, was 0.4 and 0.3 at 15 and 60 minutes post-treatment, respectively (Table 1). Although synergistic, the interaction is less profound following systemic compared to intrathecal administration.

When administered alone, morphine failed to produce >50% efficacy at either time point (15 or 60 minutes). Clonidine reached full efficacy (defined as ≥75%) at 60 but not 15 minutes following injection (Figure 1B, Table 2). In contrast, >90% efficacy was achieved by the combination at both time points (Table 2).

### Intrathecal co-administration of morphine and clonidine has an additive effect on sedation/motor impairment

Intrathecal administration of either morphine or clonidine produced dose-dependent sedation/motor impairment 15 and 35 minutes post-injection (Figure 2A, Table 1). Potency was not significantly altered following co-administration at a dose ratio of 1∶1, indicating that the interaction was additive (Figure 2A, Table 1). The interaction index, γ, was 0.9 at 15 minutes and 3.5 at 35 minutes, consistent with the lack of synergism (Figure 2A, Table 1).

When administered alone, neither morphine nor clonidine produced greater than 50% sedation 35 minutes following injection and only clonidine was fully efficacious (≥75%) at 15 minutes (Figure 2A, Table 2). Maximum efficacy was not increased by co-administration at either time point (Table 2).

### Systemic co-administration of morphine and clonidine has an additive effect on sedation/motor impairment

Systemic (i.p.) administration of either morphine or clonidine produced dose-dependent sedation/motor impairment 20 minutes post-injection (Figure 2B, Table 1) and only clonidine had efficacy (defined as >30% MPE) at 65 minutes (Tables 1,2). Co-administration at a ratio of 10∶1 did not significantly alter drug potency. While isobolographic analysis was not performed 65 minutes post-injection because one drug lacked efficacy, the ED_50_ values of clonidine alone vs. the combination were not statistically different, suggesting that the relationship between morphine and clonidine at this time-point is additive (Table 1). Maximum efficacy was not significantly altered by co-administration (Table 2).

### Intrathecal and systemic co-administration of morphine and clonidine have additive or sub-additive effects on heart rate

Intrathecal administration of either morphine or clonidine produced dose-dependent decreases in heart rate 10 and 30 minutes post-injection (Figure 3A, Table 1). Co-administration at a constant dose ratio 1∶1 did not alter drug potency (Figure 3A, Table 1). As shown in Figure 3A′, at 10 minutes post-injection the ED_50_ of the combination is not significantly different from that of the theoretical additive ED_50_, indicating that the combination has an additive effect on sedation. At 30 minutes post-injection, similar analysis revealed that the interaction was sub-additive (Table 1). The interaction indices were 1.4 and 1.7, consistent with the additive to sub-additive interactions (Table 1). Maximum efficacy was not significantly altered by drug co-administration (Table 2).

Systemic (i.p.) administration of either morphine or clonidine produced dose-dependent inhibition of heart rate 15 and 60 minutes post-injection (Figure 3B, Table 1). Co-administration at a ratio of 10∶1 resulted in a sub-additive interaction at both time points (Figure 3B′, Table 1). The interaction indices were 3.8 and 1.6 at 15 and 60 minutes, respectively, consistent with a sub-additive interaction. Maximum efficacy was not significantly altered by co-administration (Table 2).

The maximum possible effect was set at 300 beats per minute (compared to pre-drug baseline of ∼800 BPM) to facilitate isobolographic analysis. 100% MPE therefore corresponds to a decrease in BPM from 800 to 300.

### Intrathecal and systemic co-administration of morphine and clonidine have no interaction on blood pressure

**Figure 4:**
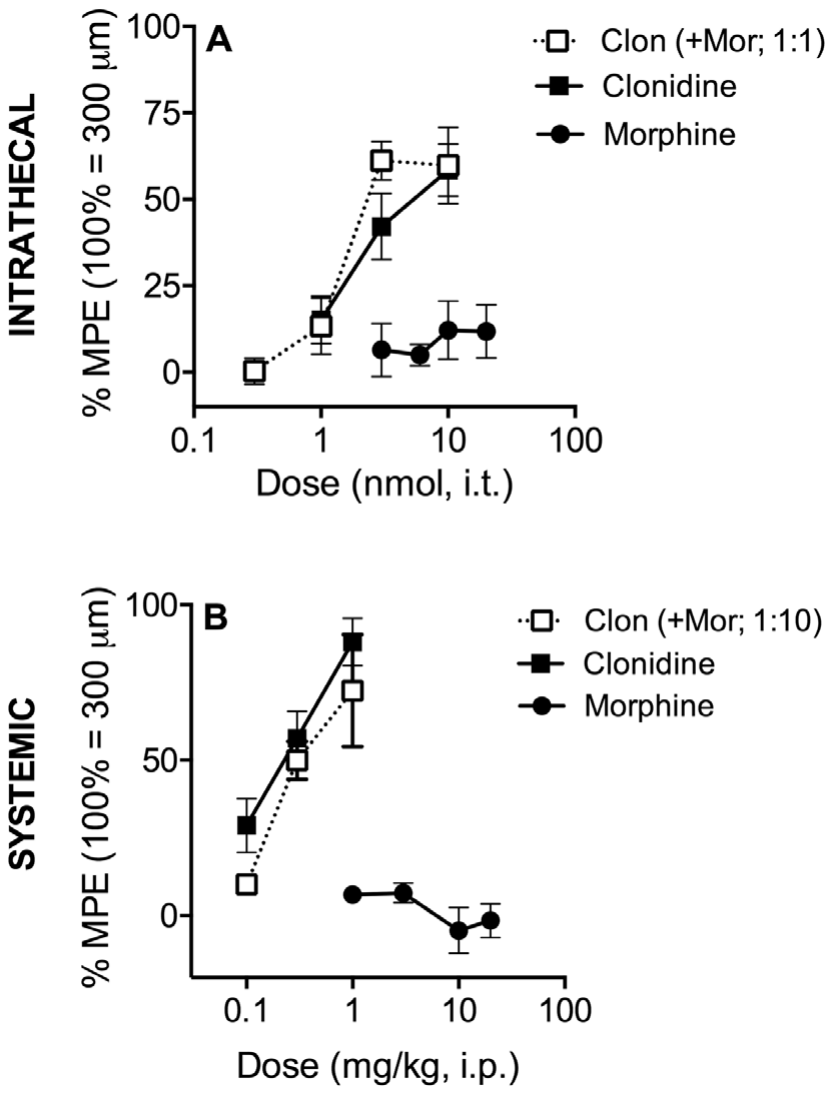
Effects of Morphine and Clonidine on Carotid Distension. Carotid distension was used as an indirect measure of blood pressure in awake, behaving animals. **A**. Carotid distension was challenged by intrathecal morphine, clonidine or both. While clonidine (▪) reduced carotid distension in a dose-dependent manner, morphine (•) was ineffective. When the agonists were co-administered at a constant ratio of 1∶1 (○ morphine; □ clonidine), the potency and efficacy of the combination was not different from that of clonidine given alone. **B**. Systemically administered clonidine (▪), but not morphine (•), reduced carotid distension in a dose-dependent manner. Neither the potency nor the efficacy of the combination of morphine∶clonidine at a dose ratio of 10∶1 (○ morphine; □ clonidine) were different from clonidine given alone. Data pictured were obtained 10 minutes following intrathecal (Figure 4A) and 15 minutes following systemic administration (Figure 4B). Error bars represent ±SEM for each dose point (*n* = 6-10 animals/dose). See Table 1 for ED_50_ values. *Note: 100% MPE was artificially set at* 300 µm for carotid distension *(baseline ∼700 µm) to facilitate dose-response and isobolographic analysis.*

Carotid distension was used as an indirect measure of blood pressure in awake, behaving animals. Whereas intrathecal administration of clonidine produced dose-dependent decreases in carotid distension 10 and 30 minutes post-injection (Figure 4A, Table 1), morphine lacked efficacy in this measure. When co-administered at a constant ratio of 1∶1, the ED_50_ value of clonidine was not statistically different from the ED_50_ value of the combination, suggesting there is no interaction (Figure 4A, Table 1). Similarly, the maximum efficacy achieved by clonidine was not affected by the presence of morphine (Figure 4A, Table 2). Isobolographic analysis was not performed because one drug lacked efficacy.

Consistent with the intrathecal results, while systemic administration of clonidine produced dose-dependent decreases in carotid distension, morphine lacked efficacy and had no effect on clonidine potency or efficacy (Figure 4B, Tables 1&2).

The maximum possible effect was set at 300 µm for carotid distension (compared to pre-drug baseline of ∼700 µm) to facilitate isobolographic analysis. 100% MPE therefore corresponds to a decrease in carotid distension from 700 to 300 µm.

### Effect of morphine and clonidine co-administration on therapeutic window

The data presented above demonstrate that co-administration of morphine and clonidine produces synergy in antinociception but not in the side effects of sedation/motor impairment or cardiovascular depression. We therefore examined the impact of co-administration on the therapeutic window (TW), a comparison of the amount of an agent required to produce the desired effect (i.e. antinociception) to the amount that produces the undesired effect (i.e. sedation, heart rate, carotid flow). Specifically, the TW is defined as the ED_50_ (undesired effect)/ED_50_ (desired effect). A TW >1 indicates that the desired effect can be achieved in the absence of the side effect and higher indices are more advantageous therapeutically.

In Table 3, the TW has been calculated for morphine and clonidine alone and in combination following intrathecal or systemic administration for each endpoint. Following intrathecal delivery, combination therapy increased the TW from between 0.5–5.7 to 36–200. These large increases in TW are the result of profound antinociceptive synergy in parallel with additive or sub-additive interactions in the undesired side effects. Systemic combination therapy resulted in more modest increases in TW compared to intrathecal delivery. This is due primarily to the magnitude of the synergistic interaction in the antinociceptive assay being greater for intrathecal vs. systemic administration.

**Table 3:** Combination Therapy Improves Therapeutic Window.

			Therapeutic Window	Therapeutic Window	Therapeutic Window
Route	Time-point	Drug(s)	Sedation/Antinociception	Heart Rate/Antinociception	Carotid Distension/Antinociception
**Spinal**	**10**–**15 min**	Clonidine	0.5	0.6	0.6
		Morphine	1.8	1.6	NA
		***Clon+Mor***	**45**	**86**	**36**
	**30**–**35 min**	Clonidine	3.8	2.1	4.0
		Morphine	5.7	2.6	NA
		***Clon+Mor***	**200**	**52**	**44**
**Systemic**	**15**–**20 min**	Clonidine	1.1	0.1	0.2
		Morphine	1.1	0.4	NA
		***Clon+Mor***	**2.2**	**1.6**	**2.0**
	**60**–**65 min**	Clonidine	2.4	0.3	0.7
		Morphine	*NA*	0.6	NA
		***Clon+Mor***	**6.8**	**1.8**	**NA**

Therapeutic Window is the ratio of the indicated ED_50_ values. Larger therapeutic windows are more advantageous therapeutically.

## Discussion

In this manuscript, the beneficial effects of adrenergic-opioid combination therapy on therapeutic window in the treatment of acute pain were determined. Significant increases in therapeutic window were observed following both intrathecal and systemic administration. The opening of the therapeutic window can be explained by the presence of a synergistic interaction in antinociception in the absence of similar potentiation in the side effects of sedation/motor impairment and cardiovascular depression. In addition to increasing the therapeutic window, combination therapy resulted in increased maximum antinociceptive efficacy. Clinically, these data suggest that improved analgesia can be obtained with combination therapy at doses that do not produce sedation or cardiovascular depression.

### Intrathecal Morphine + Clonidine

This report confirmed prior observations that the prototypic α_2_AR agonist clonidine synergizes with morphine when given spinally to mice, increasing the potency by 100-fold over either agent given alone. By contrast, the combination synergized in neither the rotarod test of sedation/motor impairment nor in measures of cardiovascular depression. The antinociceptive/sedative therapeutic window range was increased from 0.5–5.7 to 45–200 as a result of the mulitmodal vs. unimodal approach. Similarly the antinociceptive/cardiovascular therapeutic window range was increased from 0.6–4.0 to 36–86. As a result, maximum antinociceptive efficacy is obtained at doses where neither sedation/motor impairment nor cardiovascular depression is evident. This separation between antinoception/sedation or antinociception/cardiovascular depression was not observed when either drug was administered alone.

In addition to the increased therapeutic window, the maximum antinociceptive efficacy was significantly higher in the combination group than either drug administered alone. No similar increases in the maximum efficacy were observed in the sedative/motor or cardiovascular measures. Therefore, intrathecal combination therapy affords an increase in both analgesic efficacy and potency in the absence of similar increases in the undesired side effects examined in this study.

### Systemic (i.p.) Morphine + Clonidine

Following systemic administration, synergy was observed for antinociception but not in the sedative/motor or cardiovascular side-effects. However, the magnitude of the synergistic effect on antinociception was modest, yielding interaction index values of 0.3–0.4 compared to the much lower, and therefore more profound, intrathecal values of 0.02–0.07. As a result, the therapeutic window was only marginally increased by the combination. Thus, to maximize the effect of the morphine-clonidine combination therapy on therapeutic window, intrathecal delivery is advantageous.

Systemic combination therapy resulted in significant increases in the maximum antinociceptive efficacy of the combination compared to either drug alone. Thus, systemic morphine-clonidine combination therapy may result in therapeutically important increases in analgesic efficacy even in the absence of a profound impact on therapeutic window.

### Integration with pre-clinical and clinical literature

Although several studies have probed the cardiovascular interactions between α_2_AR and OR agonists [4], [34], [44], the goal of the current study was to systematically compare antinociceptive, sedative/motor and cardiovascular interactions under identical conditions (e.g. mouse strain, sex, age, housing and experimental environment). Loomis and colleagues (1988) evaluated the effects of intrathecally co-administered sub-effective doses of morphine and clonidine on sensitivity to heat (tail flick assay) and mechanical (paw pressure test) stimuli and blood pressure (tail cuff); the drug combination produced robust antinociception but had no effect on blood pressure. Solomon and Gebhart (1988) evaluated both antinociceptive and cardiovascular tolerance and cross-tolerance to the two single drugs given intrathecally, but the drugs were never co-administered. Although both drugs produced antinociceptive tolerance, chronic clonidine produced cardiovascular tolerance but unmasked a dose-related hypotensive effect of morphine. An analogous interaction on hypotension was not observed in the present study, although the current study was limited to acute drug administration. Randich and colleagues (1992) observed that a single, acute, systemic dose of morphine produced long-lived antinociception accompanied by transient (10 min) hypotension and heart rate. In the current study, while neither intrathecal nor systemic morphine effected carotid distension, they did dose-dependently reduce heart rate. Puig and colleagues (1996) performed an isobolographic analysis of systemic co-administration of clonidine and morphine and observed synergistic inhibition of gastrointestinal transit at low doses; therefore, the results reported here cannot be generalized to include all side effects [38].

In humans, studies examining the interaction between opioids and epidural clonidine in acute, post-surgical or chronic pain largely agree with our observations. For example, studies on labor or post-cesarean pain have shown that either a) clonidine reduced the need for intravenous morphine, did not affect heart rate or sedation but decreased blood pressure [13], b) clonidine improved opioid analgesia and reduced opioid self-administration in the absence of clinically important changes in side-effects [45], c) clonidine improved opioid analgesia but increased sedation [37], or d) epidural clonidine lowered fentanyl requirements during surgical anesthesia and improved cardiovascular stability with no serious untoward effects [46]. Common amongst those studies is the improved analgesic efficacy in the absence of consistent increases in the sedative and cardiovascular side effects. Furthermore, epidural clonidine has been shown to benefit patients with intractable cancer pain, particularly those with a significant neuropathic component [47], and the combination of intrathecal morphine + clonidine is useful for the management of chronic pain after spinal cord injury [17], [20].

The current data demonstrate that the combination of morphine and clonidine can yield therapeutic windows greater than those of either agent alone. Similar observations have been reported in the pre-clinical literature for the mixed opioid agonist and serotonin-norepinephrine reuptake-inhibiting effects of tramadol [48] and tapentadol [49]. In addition, the current study extends prior antinociception/cardiovascular studies [34], [50] to include full dose-response and isobolographic analysis along with the assessment of sedative/motor effects.

### Limitations and Future Directions

In the current study, sedative and cardiovascular side effects were selected for this study because they are common to both drug classes, can be a limiting factor in the therapeutic use of either drug and can be easily measured in awake, behaving mice. While sedation will result in motor impairment, factors in addition to sedation likely contribute to the treatment-related motor impairment observed in this study. Additional studies addressing other physiological effects such as the respiratory depression associated with systemic opioid agonists are necessary.

The current study was performed in acute assays in normal, drug-naive and pain-free animals. Clinically, multimodal therapies are employed in chronic pain patients that are unresponsive to other therapeutic interventions [10], [18]. While opioid-adrenergic synergy has also been demonstrated in pre-clinical models of chronic neuropathic pain [51], [52], the side-effect profiles were not simultaneously assessed. In addition, the impact of opioid tolerance [53], [54] on opioid-adrenergic synergy is not clear; for example, studies have suggested that synergy is either decreased [53] or unaltered [54] in morphine-tolerant mice. Finally, it will be important to examine these interactions during chronic administration of the combination as the rate and incidence of tolerance may develop differently in the desired vs. undesired effects, thus altering therapeutic window. If synergy-enabled reductions in total dose translate into reductions in analgesic tolerance, an important additional benefit of multimodal therapy will be realized. However, this possibility remains to be tested.

### Advantages of Multimodal Analgesic Pharmacology

The search for new analgesic agents is focused on the identification of novel drug targets and the development of highly selective compounds directed at subtypes or subunits of these targets. This quest has eclipsed the search for ways to optimize the therapeutic benefit of well-known agents with proven efficacy in patients. Recent pharmaceutical history shows that highly potent, highly selective agents, upon translation to human subjects, either lack sufficient efficacy or manifest intolerable side effects or toxicities. For example, NK-1 antagonists, COX-2 selective inhibitors and TRPV1 antagonists have all failed to meet expectations for one of the aforementioned reasons [55]–[57]. These examples suggest that the rationale underlying drug development strategies that use potency and selectively as primary criteria may be flawed. Many of the most successful analgesics historically are non-selective, including morphine and other clinically used opioids, tramadol (targets both opioid receptors and monoaminergic reuptake), cannabis, tricyclic anti-depressants and aspirin.

Building on the foundation of therapeutically validated compounds with known side-effect profiles in humans presents huge potential for development of combination therapies [58], [59]. Examples of combination medications abound, such as local anesthetics with morphine for intrathecal infusion and opioid-acetaminophen combinations for oral administration. In addition to lowering the prevalence of adverse effects and improving analgesia, multimodal analgesia techniques may shorten hospitalization times, improve recovery and function, and decrease healthcare costs [60], [61]. However, few of these combinations exploit well-characterized synergistic interactions such as the α_2_AR-opioid interaction described in this paper. Furthermore, the impact of the combination on therapeutic window is rarely considered. A systematic search of therapeutically used agents for identification of synergistic pairs and exploitation of these pairs for therapeutic development would be of great value.
